# Supplementary material for: Health-related quality of life of daily-life-affected benign essential blepharospasm: Multi-center observational study
Source: PLoS One. 2023 Mar 15;18(3):e0283111. doi: 10.1371/journal.pone.0283111 (PMC10016646; doi:10.1371/journal.pone.0283111)
Supplement: S1 Table — (DOCX) [file pone.0283111.s001.docx]

**Supplement**

**S1 Table:** Baseline Quality of Life by generic (EQ-5D-5L) and condition-specific

(NEI- VFQ25) questionnaires (Mean, SD)

| **Condition of patients** | **EQ-5D-5L** | | | **NEI- VFQ25** | |
| --- | --- | --- | --- | --- | --- |
|  | **Utility Scores** | **EQ-5D-5L**  **VAS** | **EQ-5D-5L**  **Summary** | **General health** | **Composite scores** |
| **Complete follow up (N=159)** | 0.746 ± 0.233 | 64.54 ± 19.27 | 0.252 ± 0.233 | 33.96 ± 19.95 | 58.69 ± 19.07 |
| **Sex** | | | | | |
| Female | 0.731 ± 0.239 | 63.51 ± 18.91 | 0.269 ± 0 .239 | 34.46 ± 20.22 | 57.95 ± 18.99 |
| Male | 0.869 ± 0.129 | 72.12 ± 20.71 | 0.131 ± 0.129 | 30.26 ± 17.83 | 64.14 ± 19.29 |
| **Employment** | | | | | |
| Employed | 0.776 ± 0.214 | 65.14 ± 20.27 | 0.224 ± 0.214 | 31.77 ± 20.35 | 59.52 ± 18.76 |
| Unemployed | 0.704 ± 0.256 | 63.62 ± 17.76 | 0.296 ± 0.256 | 37.30 ± 18.98 | 57.43 ± 19.61 |
| **Underlying disease (u/d)** | | | | | |
| Present of u/d | 0.723 ± 0.247 | 63.34 ± 19.39 | 0.277 ± 0.247 | 33.99 ± 20.54 | 58.37 ± 20.09 |
| No u/d | 0.810 ± 0.180 | 67.56 ± 18.84 | 0.190 ± 0.180 | 33.89 ± 18.58 | 59.51 ± 16.38 |
| **Physical severity grading by Jankovic Rating Scale (JRS)** | | | | | |
| JRS = 8, (N=48) | 0.646 ± 0.280 | 54.41 ± 21.75 | 0.354 ± 0.280 | 31.25 ± 20.30 | 48.42 ± 15.98 |
| JRS = 7, (N=32) | 0.754 ± 0.189 | 65.19 ± 19.93 | 0.246 ± 0.189 | 30.47 ± 22.66 | 57.29 ± 16.58 |
| JRS = 6, (N=79) | 0.807 ± 0.197 | 70.42 ± 14.52 | 0.193 ± 0.197 | 37.03 ± 18.28 | 65.51 ± 18.99 |

VAS= visual analog scale, JRS=Jankovic Rating Scale
