# Supplementary material for: Health-related quality of life of daily-life-affected benign essential blepharospasm: Multi-center observational study
Source: PLoS One. 2023 Mar 15;18(3):e0283111. doi: 10.1371/journal.pone.0283111 (PMC10016646; doi:10.1371/journal.pone.0283111)
Supplement: S2 Table — (DOCX) [file pone.0283111.s002.docx]

**Supplement**

**S2 Table:** Daily-life-affected BEB patients’ health-related quality of life by

EQ-5D-5L questionnaires in subscales

| **Dimension** | **Baseline N(%)** | **Follow-up* N(%)** | **p-value**** |
| --- | --- | --- | --- |
| **Mobility** | | | |
| No problems or slightly problems to walk about | 98 (62%) | 110 (69%) | < 0.001 |
| Moderate, severe or unable to walk about | 61 (38%) | 49 (31%) |  |
| **Self-care** | | | |
| No problems or slightly problems to wash or dress myself | 140 (88%) | 145 (91%) | < 0.001 |
| Moderate, severe or unable to dress myself | 19 (12%) | 14 (9%) |  |
| **Usual activities** | | | |
| No problems or slightly problems doing my usual activities | 104 (65%) | 127 (80%) | < 0.001 |
| Moderate, severe or unable to do my usual activities | 55 (35%) | 32 (20%) |  |
| **Pain/discomfort** | | | |
| No problems or slightly pain or discomfort | 99 (62%) | 116 (73%) | < 0.001 |
| Moderate, severe or extreme pain or discomfort | 60 (38%) | 43 (27%) |  |
| **Anxiety/depression** | | | |
| No problems or slightly anxious or depressed | 116 (73%) | 137 (86%) | < 0.001 |
| Moderately, severely or extremely anxious or depressed | 43 (27%) | 22 (14%) |  |

*Follow up at 1 month after botulinum toxin treatment, **McNemar’s Chi-Square test
