# Supplementary material for: Health-related quality of life of daily-life-affected benign essential blepharospasm: Multi-center observational study
Source: PLoS One. 2023 Mar 15;18(3):e0283111. doi: 10.1371/journal.pone.0283111 (PMC10016646; doi:10.1371/journal.pone.0283111)
Supplement: S3 Table — (DOCX) [file pone.0283111.s003.docx]

**Supplement**

**S3 Table:** Estimated EQ-5D-5L, NEI-VFQ-25 and JRS at each visit and coefficient with 95% confidential interval of patients with daily-life-affected blepharospasm.

| **Health Related Quality of Life** | | | | | |
| --- | --- | --- | --- | --- | --- |
|  | **Pre-treatment** | **Post treatment** | **β 4-6 weeks** | **Post treatment** | **β 12-16 weeks** |
|  |  | **4-6 weeks** |  | **12-16 weeks** |  |
| **Health-related quality of life by EQ-5D-5L** | | | | |  |
| Utility scores | 0.74 (0.72-0.77)* | 0.83 (0.80-0.86)* | 0.09 (0.05-0.11)* | 0.81 (0.78-0.84)* | 0.06 (0.04-0.09)* |
| EQ-5D-5L summary scores | 0.26 (0.23-0.28)* | 0.17 (0.14-0.20)* | -0.08(-0.11- -0.05)* | 0.19 (0.16-0.22)* | -0.06 (-0.09- -0.04)* |
| EQ-5D-5L visual analog scale | 63.60 (61.18-66.01)* | 74.84 (72.26-77.40)* | 11.24 (8.58-13.90)* | 73.41 (70.81-76.01)* | 9.82 (7.12-12.51)* |
| **Health-related quality of life by NEI-VFQ25** | | | | |  |
| General health | 34.29 (31.44- 37.15)* | 44.35 (41.32-47.38)* | 10.06 (6.79-13.33)* | 37.86 (34.79-40.93)* | 3.57 (0.25-6.88)*** |
| Composite scores | 59.53 (56.95-62.10)* | 69.54 (66.85-72.23)* | 10.01 (7.55-12.47)* | 67.15 (64.43-69.88)* | 7.63 (5.13-10.12)* |
| General vision | 61.57 (59.24-63.90)* | 69.13 (66.65-71.61)* | 7.56 (4.73-10.38)* | 66.62 (64.10-69.14)* | 5.05 (2.19-7.90)** |
| Ocular pain | 78.34 (73.40-83.27)* | 78.31 (73.18-83.43)* | -0.03 (-4.17-4.11)**** | 78.56 (73.39-83.73)* | 0.22 (-3.97-4.42)**** |
| Difficulty with near-vision activities | 55.24 (51.83-58.65)* | 69.05 (65.44-72.66)* | 13.81 (10.06-17.57)* | 65.64 (61.98-69.29)* | 10.40 (6.60-14.20)* |
| Difficulty with distance-vision activities | 57.53 (53.86-61.19)* | 68.47 (64.60-72.34)* | 10.94 (7.13-14.76)* | 65.58 (61.68-69.49)* | 8.06 (4.20-11.91)* |
| Limitation of social functioning | 61.91 (58.14-65.68)* | 73.71 (69.73-77.69)* | 11.80 (7.81-15.79)* | 69.85 (65.81-73.89)* | 7.94 (3.89-11.98)* |
| Mental health | 56.81 (53.06-60.55)* | 70.60 (66.67-74.53)* | 13.79 (10.04-17.54)* | 67.76 (63.78-71.74)* | 10.96 (7.16-14.76)* |
| Role limitations | 44.11 (40.55-47.67)* | 60.89 (57.14-64.64)* | 16.78 (13.08-20.48)* | 56.75 (52.95-60.55)* | 12.64 (8.19-16.39)* |
| Dependency | 60.30 (56.17-64.43)* | 70.45 (66.10- 74.79)* | 10.15 (5.99-14.31)* | 66.30 (61.91-70.69)* | 6.00 (1.79-10.21)*** |
| Driving difficulties | 34.74 (29.03-40.45)* | 43.26 (37.25-49.27)* | 8.52 (3.30-13.74)** | 42.24 (36.14-48.34)* | 7.50 (2.15-12.85)** |
| Difficulties with color vision | 77.23 (73.86-80.59)* | 83.52 (79.95-87.08)* | 6.29 (2.52-10.06)** | 84.24 (80.61-87.86)* | 7.01 (3.18-10.84)* |
| Difficulties with peripheral vision | 56.59 (52.68-60.49)* | 67.90 (63.77-72.02)* | 11.31 (6.97-15.65)* | 64.30 (60.11-68.50)* | 7.72 (3.31-12.12)** |
| **Physical severity grading by Jankovic Rating Scale (JRS)** | | | | |  |
| JRS severity | 3.36 (3.24-3.48)* | 0.69 (0.56-0.82)* | -2.67 (-2.83- -2.50)* | 2.56 (2.43-2.69)* | -0.80 (-0.97- -0.63)* |
| JRS frequency | 3.36 (3.24-3.48)* | 0.72 (0.59-0.85)* | -2.64 (-2.81- -2.47)* | 2.62 (2.49-2.75)* | -0.74 (-0.91- -0.57)* |
| JRS summary scores | 6.72 (6.48-6.95)* | 1.41 (1.16-1.66)* | -5.31 (-5.63-4.99)* | 5.18 (4.92-5.43)* | -1.54 (-1.86- -1.22)* |

*P-value < 0.001, **P-value < 0.01, ***P-value < 0.05, **** P-value ≥ 0.05
