# Supplementary material for: Health-related quality of life of daily-life-affected benign essential blepharospasm: Multi-center observational study
Source: PLoS One. 2023 Mar 15;18(3):e0283111. doi: 10.1371/journal.pone.0283111 (PMC10016646; doi:10.1371/journal.pone.0283111)
Supplement: Attachment — Submitted filename: Response to Reviewers.docx [file pone.0283111.s004.docx]

Dear Editors and Reviewers,

Thank you for your kind comments. I got many points of view to improve my manuscript. Here is my explanations and additional contents which already added in the revised manuscript.

**Reviewer #1:**

The authors reported an interesting study about quality of life in patients with benign essential blepharospasm (BEB). I have some comments to the authors:

- In the methods the authors should specify that the diagnosis of BEB was made in accordance with the recent criteria. Here the paper that should be included:

Defazio G, et al. Diagnostic criteria for blepharospasm: A multicenter international study. Parkinsonism Relat Disord. 2021 Oct;91:109-114. doi: 10.1016/j.parkreldis.2021.09.004. Epub 2021 Sep 8. PMID: 34583301; PMCID: PMC9048224.

**Response**:

Because we started our study on August 2020, this article hasn’t been published at that time. We used the former definition of essential blepharospasm. Then we added the diagnostic criteria and also the recent criteria in the discussion part.

Line 87-89, 108-119 (our study’s criteria)

Line 257-265 (discussion about new criteria)

In line 87-89 and 108-119

*Blepharospasm (BSP) is a form of focal dystonia characterized by excessive contractions of muscles around the eyes, especially orbicularis oculi. The spasm is interval, involuntary and unpredictable. Disease affects both motor and non-motor function including psychiatric, sleep quality, cognitive and ocular symptoms.*

*References:*

*Yang J, Shao N, Song W, Wei Q, Ou R, Wu Y, Shang HF. Nonmotor symptoms in primary adult-onset cervical dystonia and blepharospasm. Brain Behav. 2016 Dec 18;7(2):e00592. doi: 10.1002/brb3.592. PMID: 28239516; PMCID: PMC5318359.*

*Yang J, Zhang L, Hou Y, Wei Q, Ou R, Lin J, Song W, Cao B, Shang H. Sex related differences in nonmotor symptoms of patients with idiopathic blepharospasm. Sci Rep. 2021 Sep 8;11(1):17856. doi: 10.1038/s41598-021-97289-1. PMID: 34497334; PMCID: PMC8426378.*

*Novaretti N, Cunha ALN, Bezerra TC, Pena Pereira MA, de Oliveira DS, Macruz Brito MMC, Pimentel AV, Brozinga TR, Foss MP, Tumas V. The Prevalence and Correlation of Non-motor Symptoms in Adult Patients with Idiopathic Focal or Segmental Dystonia. Tremor Other Hyperkinet Mov (N Y). 2019 Feb 4;9:596. doi: 10.7916/fhnv-v355. PMID: 30783550; PMCID: PMC6377805.*

In line 257-265

*A multicenter international study revealed recent diagnostic criteria for blepharospasm. They found the stereotyped, bilateral and synchronous orbicularis oculi muscle spasms inducing eyelid narrowing closure yielded high sensitivity (96%), but only 76% specificity. If this spasm character combined with “sensory trick” and either “increased blinking” or “inability to voluntarily suppress the spasms” in the absence of a sensory trick can help increased yield for blepharospasm diagnosis (78–81% sensitivity and 83–91% specificity)*

*References:*

*Defazio G, et al. Diagnostic criteria for blepharospasm: A multicenter international study. Parkinsonism Relat Disord. 2021 Oct;91:109-114. doi: 10.1016/j.parkreldis.2021.09.004. Epub 2021 Sep 8. PMID: 34583301; PMCID: PMC9048224.*

- Please specify whether all the patients had focal dystonia. The phenomenon of spread in BEB is a frequent condition, so it would be better if the authors expanded this point in the methods. There are several studies on the topic that might be helpful:

Berman BD, et al. Risk of spread in adult-onset isolated focal dystonia: a prospective international cohort study. J Neurol Neurosurg Psychiatry. 2020 Mar;91(3):314-320. doi: 10.1136/jnnp-2019-321794. Epub 2019 Dec 17. PMID: 31848221; PMCID: PMC7024047.

Ercoli T, et al. Spread of segmental/multifocal idiopathic adult-onset dystonia to a third body site. Parkinsonism Relat Disord. 2021 Jun;87:70-74. doi: 10.1016/j.parkreldis.2021.04.022. Epub 2021 May 12. PMID: 33991781.

**Response**:

Done

Line86-87, 266-269

*Blepharospasm is an initial onset in isolated focal dystonia type which the most likely to spread, with approximately 50% risk of spread beyond the orbicularis oculi to oromandibular and neck region in 5 years after onset.*

*References:*

*Weiss EM, Hershey T, Karimi M, et al. Relative risk of spread of symptoms among the focal onset primary dystonias. Mov Disord. 2006;21:1175–1181.*

*Martino D, Berardelli A, Abbruzzese G, et al. Age at onset and symptom spread in primary adult-onset blepharospasm and cervical dystonia. Mov Disord. 2012;27:1447–1450.*

*Berman BD, Groth CL, Sillau SH, et al. Risk of spread in adult-onset isolated focal dystonia: a prospective international cohort study. J Neurol Neurosurg Psychiatry. 2020 Mar;91(3):314-320. doi: 10.1136/jnnp-2019-321794. Epub 2019 Dec 17. PMID: 31848221; PMCID: PMC7024047.*

- Among the strengths of the study, the author may also add that the Jankovic rating scale is one of the best tool to assess BEB, as recently reported in this recent international study:

Defazio G, et al. Measurement Properties of Clinical Scales Rating the Severity of Blepharospasm: A Multicenter Observational Study. Mov Disord Clin Pract. 2022 Aug 15;9(7):949-955. doi: 10.1002/mdc3.13530. PMID: 36247913; PMCID: PMC9547140.

**Response**:

Done

Line 114-115

*We use the Jankovic rating scale, worldwide for clinical research and easy to use, for assess the severity and frequency of the blepharospasm.*

*References:*

*Defazio G, Hallett M, Berardelli A, et al. Measurement Properties of Clinical Scales Rating the Severity of Blepharospasm: A Multicenter Observational Study. Mov Disord Clin Pract. 2022 Aug 15;9(7):949-955. doi: 10.1002/mdc3.13530. PMID: 36247913; PMCID: PMC9547140.*

- The results should better organize adding the p value in the text and the findings from the statistic.

**Response**:

Done

In table1, line 195, 203-205, 216-220, 223-225, 230-232, 248

- “In our study, the female to male ratio was higher than previous studies.” Please expand this point.

**Response**:

Done

Line 282-291

*In general, the female preponderance in BEB is approximately 60-71%. But we got 88% because our study included only severe cases who need botulinum toxin treatment. Prevalence of female in daily-life affected BEB are mostly the same in 14 nationwide institutes of Thailand, with median prevalence of 90% (83.92%, 95%). There has been evidence that showed higher degree of symptom severity and frequency of BEB in female than male. The use of Botulinum toxin A in BEB treatment was associated with the efficacy and cost of botulinum toxin A treatment. Male and female patients might be aware of the quality of life affected by a certain severity of BEB differently. Female patients might be more concerned of their symptoms and thus seek medical attention more than male patients do.*

*References:*

*Sun Y, Tsai PJ, Chu CL, et al. Epidemiology of benign essential blepharospasm: A nationwide population-based retrospective study in Taiwan. PLoS One. 2018 Dec 26;13(12):e0209558. doi: 10.1371/journal.pone.0209558. PMID: 30586395; PMCID: PMC6306223*

*Epidemiological Study of Dystonia in Europe (ESDE) Collaborative Group. A prevalence study of primary dystonia in eight European countries. J Neurol. 2000 Oct;247(10):787-92. doi: 10.1007/s004150070094. PMID: 11127535.*

*Defazio G, Livrea P. Epidemiology of primary blepharospasm. Mov Disord. 2002 Jan;17(1):7-12. doi: 10.1002/mds.1275. PMID: 11835433.*

*Scorr LM, Cho HJ, Kilic-Berkmen G, McKay JL, et al. Clinical Features and Evolution of Blepharospasm: A Multicenter International Cohort and Systematic Literature Review. Dystonia. 2022;1:10359. doi: 10.3389/dyst.2022.10359. Epub 2022 May 16. PMID: 36248010; PMCID: PMC9557246.*

*Lee MS, Johnson M, Harrison AR. Gender differences in benign essential blepharospasm. Ophthalmic plastic and reconstructive surgery. 2012;28(3):169-170.*

*Snir M, Weinberger D, Bourla D, Kristal-Shalit O, Dotan G, Axer-Siegel R. Quantitative changes in botulinum toxin a treatment over time in patients with essential blepharospasm and idiopathic hemifacial spasm. Am J Ophthalmol. 2003 Jul;136(1):99-105. doi: 10.1016/s0002-9394(03)00075-8. PMID: 12834676.*

*Chundury, Rao V. M.D., M.B.A.*; Couch, Steven M. M.D.†; Holds, John B. M.D.*‡§. Comparison of Preferences Between OnabotulinumtoxinA (Botox) and IncobotulinumtoxinA (Xeomin) in the Treatment of Benign Essential Blepharospasm. Ophthalmic Plastic and Reconstructive Surgery: May/June 2013 - Volume 29 - Issue 3 - p 205-207 doi: 10.1097/IOP.0b013e31828de0d6*

**Reviewer #2:** The authors present data about quality of life of Thai patients with blepharospasm treated with Botulinum toxin type A. The manuscript might have important clinical message and may be of great interest to the readers who are interested in this specific topic.

However, I do believe that the manuscript would benefit from a second round of major revisions in order to improve it before being considered for publication. Below are some recommendations:

Q1. Line 63: “Most of the patients had bilateral involvement (96.9%)”

Please discuss why you consider few patients with unilateral involvement as having blepharospasm rather than hemifacial spasm taking into account that all more accepted definitions describe blepharospasm as a bilateral disturb.

**Response**:

Done

Line 262-271

*In the study, we have 5 unilateral blepharospasm patients. All of them are in first 3 years after onset. These can progress to bilateral blepharospasm, or hemifacial in the future. But in the recruitment time, we recruited they had only involuntary spasm of the orbicularis oculi with sensory trick, no abnormal contraction of lower face. However, blepharospasm is the most likely to spread compare with other type of focal dystonia, with approximately 50% risk of spread beyond the orbicularis oculi over 5 years.*

*References:*

*Grandas F, Elston J, Quinn N, Marsden CD. Blepharospasm: a review of 264 patients. J Neurol Neurosurg Psychiatry. 1988 Jun;51(6):767-72. doi: 10.1136/jnnp.51.6.767. PMID: 3404184; PMCID: PMC1033145.*

*Weiss EM, Hershey T, Karimi M, et al. Relative risk of spread of symptoms among the focal onset primary dystonias. Mov Disord. 2006;21:1175–1181.*

*Martino D, Berardelli A, Abbruzzese G, et al. Age at onset and symptom spread in primary adult-onset blepharospasm and cervical dystonia. Mov Disord. 2012;27:1447–1450.*

Q2. Line 97-98 : “We evaluated HRQOL in Thai patients who had daily-life-affected BEB before and after…” What do you mean by “daily-life-affected” when you consider a disease with continuous symtpoms like blepharospasm? Do you refer to more severe forms of BEB? Please specify

**Response**:

Done, already added on

Line 99-101, 108-113

*The “daily-life-affected” BEB refers to patients with severe blepharospasm symptoms that affect their normal routine daily life or work. Then the inclusion criteria were JRS at least grade 3 in both severity and frequency assessment. The results of the study might provide supporting evidence for amending the Nation’s reimbursement policy for daily-life-affected BEB treatment.*

Q3. Lines 107-108 “Inclusion criteria were daily-life-affected BEB patients who had Jankovic rating scale (JRS) of at least 3 in both severity and frequency scores graded…”

Please provide a brief sentence on the scoring graduation of the Jankovic rating scale (for istance : The JRS ranges from 0 to 8 points (sum score) and includes 2 categories:… etc etc). Moreover, a score of at least 3 for both severity and frequency on the Jankovic rating scale is relatively high as compared to those present in several case series from the literature (see works by Jankovic J, et al. 10.1002/mds.22368; and Jankovic J, et al. 10.1002/mds.23658), where Mean JRS sum score was less than 6 despite of a disease duration of more than 6 years (5.05 ± 4.25 years in the present study). It would be of great interest to report which rate of patients from the 14 centers across Thailand reached a Jankovic rating scale total score more than 6. Had all patients higher severity scores? why was the severity of the blepharospasm in this study population higher than that of the general population (as you stated – line 243)? The blepharospasm is a disorder almost exclusively managed in tertiary centers worldwide and this should not be a reason why the severity scores are so high in BEB patients in this study. Consider this in the discussion.

**Response**:

Because of the use of BTX-A for BEB is not covered by Thai universal health care coverage because there has been no previous study investigating the cost and benefits of BTX-A injection for BEB in Thai population. We evaluated HRQOL in Thai patients who had daily-life-affected BEB before and after BTX-A treatment, including efficacy and safety of the treatment. The data from this study will also use in economic evaluation (cost utility) to consider reimbursement for the blepharospasm patients who had severe symptoms which affected normal routine daily-life activities or working. Then we selected only severe group (sum score ≥ 6) first for reimburse health coverage from the government because they need help.

For the rate of severity, we didn’t collect the data from this study. But it’s very interesting, thank you for initiating great idea to collect our national database.

We explained in Line 94-101, 108-112, 186-188

Q.4 Line 130: “Both versions of the Thai questionnaires were validated in previous studies”. please report reference of the previous studies were a the Thai version of the 25-item National Eye Institute visual function questionnaire (NEI VFQ-25) was validated.

**Response**:

Done and the article of Validation Thai-version of NEIVFQ-25 and EQ5D5L are in Thai journal.

Line144

Q5. Line 179: “Table 1: Demographic data of patients with daily-life-affected BEB”

Please specify in the table that JRS scores refer to baseline state.

**Response**:

Done

Line 194

Q6. Lines 221-222-223 : “Duration of action analyzed by Kaplan-Meier estimates, 50% of participants reported the beginning of recurrent BEB symptoms at 10 weeks after the treatment.”

Please give sense to the first part of the sentence.

**Response**:

Done

Line 242-246

*Duration of action is defined as time from BTX-A injection to the time participants started to feel eyelid spasm. We found that 50 percent of participants reported that they started to feel the beginning of blepharospasm at 10th week after treatment (i.e., median duration of action is approximately 10 weeks).*

Q7. the General health item and composite score of the 25-item National Eye Institute visual function questionnaire (NEI VFQ-25) are first described in the results and discussion. It would be better to introduce them in the methods firstly. In particular, unlike the other scores, Composite scores increase after treatment, so it should be better defined in methods section.

**Response**:

Done

Line 137-140

Q8. Figures:

most Y-X graphs’ legends are almost unreadable. Please increase graphs’ quality or limit the number of graphs/figures in order to increase size (fig. 4 is a good solution)

**Response**:

I already made new figures and checked with PACE before uploaded all new figures.

Q9. Statistical analysis and results:

It would be more appropriate to specify coefficient and significance values when comparing pre and posttreatment data (both in text and tables)

**Response**:

Done

Line 203-205, 216-220, 223-225, 230-232, 248 and supporting information S3

**Reviewer #3:** This research is an accurate description of demographic and clinical features of BEB in Thailand. Authors show with adeguate statistical tools how quality of life improves after treatment with botulinum toxin. Authors used different and validated scales with reliable results.

**Response for all reviewers**:

Thank you very much for your kind reviews. Your comments are very useful for us to improve our manuscript.
