# Supplementary material for: Health-related quality of life of daily-life-affected benign essential blepharospasm: Multi-center observational study
Source: PLoS One. 2023 Mar 15;18(3):e0283111. doi: 10.1371/journal.pone.0283111 (PMC10016646; doi:10.1371/journal.pone.0283111)
Supplement: Attachment — Submitted filename: Response to Reviewers2_190223.docx [file pone.0283111.s005.docx]

Dear Editors and Reviewers,

Thank you for your kind comments. I got many points of view and grammar to improve my manuscript. Here is my explanations and additional contents which already added in the 2^nd^ revised manuscript.

**Additional Academic Editor Comments:**

In addition to the comments of our eminent referees, please consider the following:

- Line 63: add p-values.

>> Done (p<0.001)

- Line 77: change "depends of multifactor" with "are multifactorial".

>> Done

- Line 78: change "another" with "one of".

>> Done

- Line 86: change "that" with "whose".

>> Done

- Line 108: remove the sentence from "we would like..." to "... then the". Start with inclusion and exclusion criteria, which should be reported in a more systematic way.

>> Done

- Line 121: change "was pregnant" with "were pregnant".

>> Done

- Line 125: explain what is the most common "standard routine" used in your centers.

>> Done. Change to “All participants received BTX-A treatment from each center to control the spasm, and the dose depended on the severity, previous response and type of BTX-A.”

- Line 178 and 185: change "underlying diseases" with "comorbid diseases" or "comorbidity".

>> Done

- Line 188: I would not say "significant number" if the percentage is 12.5%.

>> Done.

- Line 190: what do you mean by "transportation cost"? And why is it reported?

>> In this project, we plan to use the information from this study to do the economic analysis in the future, then we collect cost of transportation from the patients. In this article, we showed about QOL in patients with daily-life affected blepharospasm and improvement after Botulinum toxin treatment. Then, we removed the cost of transportation from this article, in results and in the table 1.

- Line 196-198: acronyms should be listed in alphabetical order.

>> Done

- Line 203-205: why are you reporting the predicted scores using the linear mixed model? What is the meaning to give to the predicted scores, compared to the actual scores? Similarly for the other reported scores (line 219, etc.)

>> We did not aim to compare the predicted score to the actual scores. Since the quality-of-life scores were measured repeatedly in three visits within each patient. Thus, the data for each patient is correlated. To adjusted for correlated data, we use a linear mixed model to estimate the scores and we called the scores estimated from the linear mixed model as a predicted score. We just would like to show the value of scores after adjusting for the correlated nature of the data.

- Line 214: remove the opening parenthesis.

>> Done

- Line 257-262: move the paragraph from "a recent multicenter..." to "... and specificity [27]" in the limitations section of the article.

>> Already moved and rearranged to line 315-321

- Line 262-291: move the sentences after the discussion of your results. The discussion section should be focused on the results of YOUR paper, and then a comparison with previous literature.

>> Already rearranged in line 258-279

- Line 318: start a new paragraph when reporting the strength and limitations of your paper. Another limitation is the fact that some interview (how many?) were performed by remote, due to COVID19 pandemia.

>> Already restarted paragraph of strength and limitations. Also rewrite in some part of the limitation 315-330

- In the introduction it is said that the treatment with BTX-A is not covered by your health system. How do you treated the patients included in your study? Do they payed the treatment? This could also be a selection bias, that should be included in the limitations section.

>> Done. Already added in limitation. “The new patients who self-pay their medical expenses might deny Botulinum toxin treatment due to financial limitation. They, thus, were not including into the study. This resulted in another potential selection bias.” Line 322-4

**Reviewer #2:**

Reviewer #2: The authors aswered to most of the comments. However, I have further recommendations in order to get acceptable english language. Please check the following sentences:

1. line 86: “Blepharospasm is one of focal dystonias that symptoms commonly spread to…”

>> Done. Blepharospasm is one of focal dystonias whose symptoms commonly spread to… as editor suggested.

2. lines 114-115 : “We use the JRS, worldwide for clinical research and easy to use [21], for assess the severity and frequency of the blepharospasm”

>> Done. We use the JRS, which is widely used for clinical research and is easy to use [21], to assess the severity and frequency of blepharospasm.

3. line 288 : “Male and female patients might aware of the…”

>> Done. Male and female patients may perceive the affected quality of life differently, even if the severity is the same. Line 280-281

4. lines 301-302 : “Mental health and difficulties with vision activities in near, distance and peripheral had effects…”

>> Done. From NEI-VFQ25, mental health, difficulty with near-vision activities, distance-vision activities and peripheral vision had effects in the 50-60% range. Line 293-4

5. line 316: “BTXA treatment is useful for decreased physical severity…”

>> Done: BTX-A treatment not only reduces physical severity but also enhances quality of life, including in non-physical aspects. Line 308-9

6. lines 320-322 : “The large network of cooperation from participating neuro ophthalmologists and neuro-medicine units to deploy both generic and visual-specific questionnaires allowed for broader data collection however, this study did have some limitations”

>> Done. The strength of this study is that we conducted a prospective multicenter, observational study from neuro-ophthalmologists and neurologists in 14 centers nationwide with large population that included participants from all parts of Thailand. Data on quality of life, collected through generic and visual-specific questionnaires, broadened the scope of data collection. Line 311-4

Moreover, Martino et al., 2012 and Weiss et al.,2006 references are reported twice in refercences list. (9-30 and 8 – 29 , respectively); Yang et al., 2021 reference is reported three times (5-7-12);

>> Thank you very much for your information. I already revised all the references.

**Response for all reviewers**:

Thank you very much for your kind reviews. Your comments are very useful for us to improve our manuscript.
